# Novel TTN Mutation Causing Severe Congenital Myopathy and Uncertain Association with Infantile Hydrocephalus

**DOI:** 10.1155/2023/5535083

**Published:** 2023-07-18

**Authors:** Palanikumar Balasundaram, Indirapriya Darshini Avulakunta, Leslie Delfiner, Paul Levy, Katie R. Forman

**Affiliations:** ^1^Division of Neonatology, Jack D. Weiler Hospital, The Children's Hospital at Montefiore and Albert Einstein College of Medicine, Bronx, NY, USA; ^2^Division of Neurology, The Children's Hospital at Montefiore and Albert Einstein College of Medicine, Bronx, NY, USA; ^3^Division of Medical Genetics, The Children's Hospital at Montefiore and Albert Einstein College of Medicine, Bronx, NY, USA

## Abstract

Arthrogryposis multiplex congenita (AMC) is characterized by nonprogressive symmetric contractures of multiple joints with normal intellect and normal systemic examination. AMC is often due to fetal akinesia, which has neurologic, muscular, and connective tissue etiologies. We present a case of AMC due to a variant in the titin (TTN) gene in a term neonate. The infant is homozygous for this variant, *c.38442dup*, which is predicted to result in a truncated protein (*p.Pro12815Thr fs*∗*37, NM_001267550.2*). A literature search (PubMed) failed to find reports of this TTN variant. The variant was classified as pathogenic and submitted to ClinVar. Titin is the body's largest protein, expressed in skeletal and cardiac muscles and encoded by the TTN gene. Due to its large size (364 exons), the TTN gene has been difficult to sequence; the number of variants in the TTN gene and the spectrum of titinopathies are probably underestimated.

## 1. Case Presentation

A male neonate was born at term via elective cesarean section to a 35-year-old woman in her fourth pregnancy (gravida 4) and had a history of two prior spontaneous abortions and one termination of pregnancy. Second-trimester fetal anomaly scan revealed oligohydramnios and suspected fetal arthrogryposis with hyperflexed akinetic arms and rocker-bottom feet. A fetal MRI brain was normal. The mother had negative carrier screening for pathogenic variants in two known arthrogryposis-related genes (*SLC35A3* and *GLE1*).

At birth, the neonate emerged hypotonic with poor respiratory effort that required continuous positive airway pressure (CPAP) followed by noninvasive positive pressure ventilation. APGAR scores were 6, 6, and 7 at 1, 5, and 10 minutes, respectively. He was admitted to NICU for further management. His admission growth parameters were weight 2.585 Kg (∼5th percentile), head circumference 36 cm (∼90th percentile), and length 50 cm (∼50th percentile) using WHO growth curves.

## 2. Physical Exam

On examination, the neonate had overt facial paralysis with limited lateral eye movement, a depressed nasal bridge, floppy tongue, high-arched palate, tenting of the upper lip, a long uvula, bell-shaped chest, pectus excavatum, minimal palmar creasing, and bilateral rocker-bottom feet. There was no palpable organomegaly. Both testes were undescended, and the anus was posteriorly displaced. He was awake, alert, reactive to touch, and had spontaneous eye-opening. Axial tone was markedly decreased, and distal muscle bulk was reduced. There was overt weakness and areflexia. He had proximal joint contractures of the right upper and bilateral lower extremities. The left upper extremity was flaccid and without contracture initially.

## 3. Clinical Course

### 3.1. Respiratory

The neonate was placed on conventional mechanical ventilation for insufficient respiratory effort. Efforts to wean him were not successful and he had a tracheostomy placed on day 44 of life for continued management of his airway.

### 3.2. Cardiovascular

An initial echocardiogram showed a patent foramen ovale with high right-ventricular systolic pressure which improved in follow-up echocardiograms.

### 3.3. Nutrition

The neonate was started on total parental nutrition, which was discontinued on day of life (DOL) 14 when full enteral feeding volume was reached via orogastric tube. Liver structure was normal on ultrasound and liver function testing remained normal. He did not display any significant electrolyte imbalances. He continued to have a weak and uncoordinated suck. A gastrostomy tube was placed on DOL 44 for enteral nutrition.

### 3.4. Hematology

The infant required two transfusions during his NICU stay for dropping hemoglobin levels (lowest hemoglobin was 8.6 g/dL).

### 3.5. Urogenital

He required a urinary catheter from DOL 3–14, at which point his spontaneous voiding normalized. Scrotal ultrasound showed the left testicle to be in the inferior aspect of the pelvis and the right testicle at the superior aspect of the inguinal canal. Renal and kidney ultrasounds were normal. His blood urea nitrogen and creatinine remained normal for age.

### 3.6. Endocrine

In view of bilateral cryptorchidism and newborn screen suggesting hypothyroidism, an endocrinology evaluation was completed and was normal (Table 1).

### 3.7. Genetics

The provisional diagnosis was arthrogryposis multiplex. There was concern for muscular etiology given the normal fetal MRI of the brain and decreased muscle mass. Spinal muscular atrophy was excluded. A broad neuromuscular disorders panel was sent and revealed a homozygous mutation of the titin gene TTN: *c.38442dup*, (*p.Pro12815Thr fs*∗*37*), which was supportive of a diagnosis of congenital titinopathy. The homozygous mutation for a sequence variant in the TTN gene *c.38442dup* has resulted in a truncated protein (*p.Pro12815Thr fs*∗*37*) with abnormal function.

### 3.8. Neurological

**Figure 1:**
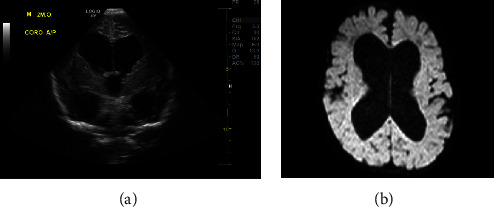
Head ultrasound (a) and MRI brain (b) showing a new finding of diffuse marked ventriculomegaly suggestive of communicating hydrocephalus.

MRI on DOL 17 revealed normal brain parenchyma and an intact corpus callosum. On DOL 75, it was noted that the head circumference had increased 3 cm over the previous week. Head ultrasound showed severe ventriculomegaly with dilatation of all the ventricles. MRI of the brain confirmed the new finding of diffuse marked ventriculomegaly suggestive of communicating hydrocephalus (Figure 1). Right-sided ventriculoperitoneal shunt placement was performed. Electromyography and muscle biopsies were deferred in this case as the genetic testing resulted quickly in providing a diagnosis.

### 3.9. Musculoskeletal

**Figure 2:**
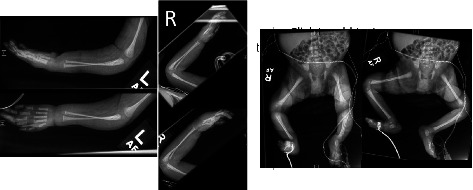
Skeletal survey of upper extremity and lower extremity showing thin and gracile bones with decreased muscle mass.

A skeletal survey of the upper and lower extremities revealed thin gracile bones and decreased muscle mass (Figure 2).

At 214 DOL, the infant was transferred to a rehabilitation center. He was subsequently transferred to a pediatric intensive care unit in view of pyelonephritis with obstructing right nephrolithiasis and right-sided hydronephrosis. He received antibiotics and a right percutaneous nephrostomy tube followed by a ureteral stent. At a year of age, he has shown little improvement and is still ventilator dependent and continues to be fed through his gastrostomy tube. He receives ongoing physiotherapy and occupational therapy services for his contractures. He requires a proactive airway clearance regimen.

## 4. Discussion

Congenital myopathies are a group of heterogeneous genetic muscle disorders characterized by hypotonia and weakness presenting at birth. The estimated prevalence is 1 in 26,000 and constitutes about 14% of all cases of neonatal hypotonia [1, 2]. There are many different subtypes of congenital myopathy, which include the nemaline myopathies, central core myopathy, centronuclear myopathy, myotubular myopathy, multimini core disease, congenital fiber-type disproportion myopathy, myosin storage myopathy, and many others. The features of specific disorders and the spectrum of severity are broad. Prenatal weakness can lead to akinesia, polyhydramnios, and arthrogryposis. Our infant presented prenatally with fetal akinesia, arthrogryposis, and had severe myopathy resulting in respiratory failure. The pattern of this child's examination was most suggestive of neuromuscular etiology.

Arthrogryposis multiplex congenita (AMC) is characterized by nonprogressive symmetric contractures of several joints starting at birth with normal intellect and normal systemic examination [3, 4]. The incidence of AMC is 1 in 5,000 live births with 30% having a genetic etiology [5]. Of all the causes of AMC, amyoplasia is the most common. The lack of digital creases in our neonate suggested no movement from very early on during development. Fetal akinesia sustained for more than three weeks can cause contractures at the time of birth [3]. Transient neonatal myasthenia, congenital myasthenic syndromes, disorders of nerve, and congenital myotonic dystrophy were considered and tested for, but were less likely. Congenital myopathy was the leading diagnosis for the neonate, given the clinical picture.

Our infant had duplication of the TTN gene at position *c.38442* on chromosome 2q31.2, which is expected to cause a frameshift, resulting in the production of a truncated protein (*p.Pro12815Thr fs*∗*37, NM_001267550.2*). The *c.38442dup* variant was confirmed with Sanger sequencing using unique PCR primers. PubMed literature search failed to find any reports of this homozygous variant. A similar, nearby homozygous deletion in this region has been reported in an infant with arthrogryposis and congenital titinopathy [6].

TTN mutations can cause a wide spectrum of muscle disorders called titinopathies. Titin is the largest protein in the body expressed in both skeletal and cardiac muscles and is encoded by the TTN gene, which has 364 exons (the first noncoding exon and 363 coding exons) [7]. The titin gene transcripts demonstrate an intricate splicing pattern characterized by multiple well-established isoforms, including N2A in skeletal muscle and N2B and N2BA in cardiac muscle [7, 8]. Novel TTN mutations are detected by targeted next-generation sequencing (NGS), prior to which it was impossible to study the TTN gene because of its complexity and large size [9]. Only a few TTN mutations have been reported because of this reason, and the spectrum of titinopathies is probably underestimated.

Autosomal dominant titinopathy is typically adult-onset, and the spectrum includes late-onset tibial muscular dystrophy, hereditary myopathy with early respiratory failure and dilated cardiomyopathy. Autosomal recessive titinopathy is a prenatal or infant-onset severe form of axial‐predominant congenital myopathy and has even been associated with a rare form of lethal congenital contracture syndrome [10]. Axial involvement includes neck weakness, chest wall deformities, scoliosis, and respiratory insufficiency. Axial weakness progresses rapidly, and limb weakness is usually nonprogressive. The most common cause of mortality is respiratory insufficiency. More than 50% of the cases will have cardiac abnormalities, including dilated cardiomyopathy [7]. Our infant does not have cardiomyopathy; however, continues to be screened monthly for progression.

The advent of next-generation sequencing has led to a remarkable surge in the discovery of novel TTN variants. Meta-transcript-only pathogenic variants of the TTN gene are exclusively identified in individuals affected by congenital titinopathy. These patients commonly exhibit prenatal-onset weakness and antenatal arthrogryposis multiplex congenita. The disease generally presents as a gradually progressive condition in individuals with at least one alle carrying a variant in meta-transcript-only exons [11]. Several recent reports have documented autosomal recessive congenital titinopathies associated with protein-truncating variants occurring exclusively in meta-transcript-only exons [10, 12–16]. Fetuses harboring meta-transcript-only variants exhibit a severe phenotype characterized by developmental anomalies such as dysgenesis of the corpus callosum, congenital bone fractures, and hypoplastic heart, resulting in the highest rate of fetal lethality [17]. Homozygous recessive mutations within the N-terminal domain of the TTN gene lead to a lethal outcome and biallelic TTN variations are also associated with severe congenital titinopathy [6, 15].

In this case report, the patient exhibits a homozygous mutation in the TTN gene, specifically the *c.38442dup* sequence variant located within exon 195, an exclusive exon found in meta-transcript isoforms. This mutation causes a premature stop codon in isoforms incorporating this exon, producing a truncated protein (*p.Pro12815Thr fs*∗*37*) with impaired functionality. The presence of the truncated protein in the fetal skeletal isoform is likely responsible for the development of severe antenatal myopathy. It is worth emphasizing that the cardiac muscle remains unaffected due to the absence of this exon in all cardiac titin isoforms (https://www.cardiodb.org/titin/titin_transcripts.php).

The new finding of communicating hydrocephalus in our neonate with congenital titinopathy has not previously been reported. Currently, it is unclear if the development of hydrocephalus is associated with congenital titinopathy.

## 5. Conclusions

In conclusion, TTN mutations should be considered for infants presenting with fetal akinesia and myopathy. This report of novel mutations expands the mutation spectrum of the TTN gene. The clinical spectrum of disorders associated with changes in the titin gene (TTN) is broad; this case identifies a phenotype on the severe end of the clinical spectrum. The phenotypic presentation of infantile hydrocephalus could be considered in children with mutations in the TTN gene. It may be reasonable to perform serial head circumference measurements in children who have mutations of the TTN gene to screen for possible hydrocephalus.

## Figures and Tables

**Table 1:** Endocrine work up.

Test	Results	Interpretation
ACTH stimulation test-cortisol (*μ*g/dl)	<1 (pre) ⟶ 24.8 (post)	
ACTH (pg/ml)	17 (not elevated)	No adrenal insufficiency
FSH (mIU/mL)	2.77	
LH (mIU/mL)	6.23	Appropriate for mini puberty
Comprehensive steroid panel	Normal	
Thyroid function test	TSH 5.6 *μ*U/ml, free T4 1.2 ng/dl	Normal

## Data Availability

No data were used in this study.
